# Cholecystectomy and sphincterotomy in patients with mild acute biliary pancreatitis in Sweden 1988 - 2003: a nationwide register study

**DOI:** 10.1186/1471-230X-9-80

**Published:** 2009-10-23

**Authors:** Birger Sandzén, Markku M Haapamäki, Erik Nilsson, Hans C Stenlund, Mikael Öman

**Affiliations:** 1 Department of Surgical and Perioperative Sciences; Surgery, Umeå University Hospital, SE-901 85 Umeå, Sweden; 2 Department of Public Health and Clinical Medicine; Epidemiology and Public Health Sciences, Umeå University, SE-901 87, Umeå Sweden

## Abstract

**Background:**

Gallstones represent the most common cause of acute pancreatitis in Sweden. Epidemiological data concerning timing of cholecystectomy and sphincterotomy in patients with first attack of mild acute biliary pancreatitis (MABP) are scarce. Our aim was to analyse readmissions for biliary disease, cholecystectomy within one year, and mortality within 90 days of index admission for MABP.

**Methods:**

Hospital discharge and death certificate data were linked for patients with first attack acute pancreatitis in Sweden 1988-2003. Mortality was calculated as case fatality rate (CFR) and standardized mortality ratio (SMR). MABP was defined as acute pancreatitis of biliary aetiology without mortality during an index stay of 10 days or shorter. Patients were analysed according to four different treatment policies: Cholecystectomy during index stay (group 1), no cholecystectomy during index stay but within 30 days of index admission (group 2), sphincterotomy but not cholecystectomy within 30 days of index admission (group 3), and neither cholecystectomy nor sphincterotomy within 30 days of index admission (group 4).

**Results:**

Of 11636 patients with acute biliary pancreatitis, 8631 patients (74%) met the criteria for MABP. After exclusion of those with cholecystectomy or sphincterotomy during the year before index admission (N = 212), 8419 patients with MABP remained for analysis. Patients in group 1 and 2 were significantly younger than patients in group 3 and 4. Length of index stay differed significantly between the groups, from 4 (3-6) days, (representing median, 25 and 75 percentiles) in group 2 to 7 (5-8) days in groups 1. In group 1, 4.9% of patients were readmitted at least once for biliary disease within one year after index admission, compared to 100% in group 2, 62.5% in group 3, and 76.3% in group 4. One year after index admission, 30.8% of patients in group 3 and 47.7% of patients in group 4 had undergone cholecystectomy. SMR did not differ between the four groups.

**Conclusion:**

Cholecystectomy during index stay slightly prolongs this stay, but drastically reduces readmissions for biliary indications.

## Background

The annual incidence of first attack of acute pancreatitis varies from below 10 to over 40 per 100000 inhabitants per year [1]. In population-based studies one-fourth to one-half of all cases with first attack acute pancreatitis is attributable to gallstone disease [1-3]. Whereas patients with acute biliary pancreatitis and coexisting acute cholangitis benefit from early endoscopic sphincterotomy [4], early endoscopic retrograde cholangiopancreatography (ERCP) has not been shown to lead to a significant reduction in overall morbidity and mortality in predicted mild or predicted severe biliary pancreatitis without acute cholangitis [5].

In the UK guidelines it is stated that patients with mild to moderate acute pancreatitis of gallstone origin should undergo definitive management of gallstone disease during the same hospital admission, unless a clear plan has been made for definitive treatment within the next two weeks [6]. Similar recommendations have been given by the International Association of Pancreatology [7]. However, according to US guidelines, expectant management with interval cholecystectomy is appropriate for most patients with mild to moderate pancreatitis and an improving clinical course [8]. We have analyzed patients with mild acute biliary pancreatitis (MABP) treated with cholecystectomy/sphincterotomy within 30 days of index admission and the effect on readmission rate, total cholecystectomy rate at one year, and mortality at 90 days. Data from nationwide hospital discharge and death certificate registers were used. It was considered of particular interest to compare outcome after cholecystectomy during index stay with cholecystectomy after index discharge but still within one month of index admission.

## Methods

In Sweden patients are identified by a national registration number unique for each resident in Sweden. The Swedish National Board of Health and Welfare's Epidemiology Centre compiles data on individual hospital discharges in the National Patient Register (NPR) [9]. Since 1987 the register has included all Swedish hospitals. The record of each hospital stay contains diagnoses at discharge coded according to the Swedish version of the International Classification of Diseases (ICD). ICD from 1987 through 1996 entailed the 9^th ^revision and from 1997 the 10^th ^revision. Hospital stays are classified as emergent or elective. Surgical procedures are coded according to the Swedish version of Classification of Operations 1988 and Classification of Surgical Procedures 1997.

For all records reported to NPR, the data are checked for authenticity. A quality control is conducted to confirm that different variables and dates are and that compulsory variables (personal identification number, hospital and main diagnosis) are reported. Obviously incorrect data are corrected. In 2003, 1.0% of all diagnoses and 0.5% of acute somatic diagnoses were missing in the hospital stays reported [9].

For the present study, data from NPR and from the death certificate register were retrieved for all patients with a hospital admission from 1987 through 2004 for acute pancreatitis (ICD-9: 577A; ICD-10:K85) and biliary disease (ICD-9: 574, 575, 576; ICD-10: K80, K81, K82, K83) at index admission.

MABP was defined as acute pancreatitis for biliary disease without mortality during an index stay of ten days or shorter. Information on hospital admissions one year prior to index admission was collected to exclude patients with recurrent pancreatitis. When readmitted after index admission, recurrent acute pancreatitis is included in biliary diagnoses.

Patients with MABP were classified into four groups. Group 1 were those with cholecystectomy during index stay and group 2 those in which cholecystectomy was not performed during index stay but still within 30 days of index admission (scheduled or unscheduled readmission), and group 3 those with sphincterotomy but not cholecystectomy within 30 days of index admission (at index admission or at early readmission). In group 4 neither cholecystectomy nor sphincterotomy was performed within 30 days of index admission.

### Procedure codes used

Open cholecystectomy: 5350, 5351, 5352, 5356, 5357, 5359, JKA20.

Laparoscopic cholecystectomy: 5353, JKA21.

Sphincterotomy: 5388, 5394, JKE02

### Statistics

Data are presented as median values with 25% and 75% percentiles, means and standard deviations or proportions. Proportions were compared using the chi-square test or Fisher-exact test when appropriate. Location of two or more groups of ratio scale variables are compared using Anova/T-test or Kruskal-Wallis/Mann Whitney test. Post hoc tests are done using Bonferroni adjustment of p-values. Mortality within 90 days of index admission was presented as case fatality rate (CFR, deaths per 100 patients) and standardized mortality ratio (SMR), using age-, gender-, and calendar year-specific expected survival estimates from Statistics Sweden [10]. SMR is presented as mean with 95% confidence intervals. Calculations were performed with SPSS 15.0 (SPSS Inc. Chicago, IL, USA). A p-value less than 0.05 was considered significant.

## Results

From 1 January 1988 through 31 December 2003, 8631 of 11363 patients with acute biliary pancreatitis fulfilled our criteria for MABP. After exclusion of 212 patients with cholecystectomy or sphincterotomy during the year preceding index admission, 8419 patients with MABP remained for analysis (74.1% of all patients with acute biliary pancreatitis, 3296 men and 5123 women).

Table 1 illustrates age and outcome of patients with biliary diagnosis including recurrent acute pancreatitis in each group within one year from index admission. Age of patients in the four groups differed significantly. Patients in groups 1 and 2 were significantly younger than patients in groups 3 and 4 (p < 0.001). Those who had cholecystectomy during index admission (group 1) had a modest, but statistically significant, longer index stay, i.e. length of hospital stay (LOS), compared to patients in groups 2 to 4 (p < 0.001). Only 4.9% of patients in group 1 returned for at least one further hospital admission with a biliary diagnosis including acute pancreatitis within one year of index admission, compared to 62.5% of patients in group 3, and 76.3% of patients in group 4.

**Table 1:** Characteristics of the four different MABP groups

	Group 1	Group 2	Group 3	Group 4	All groups
**Number of patients**	834 (9.9%)	804 (9.6%)	977 (11.6%)	5804 (68.9%)	8419 (100%)
**Age, years. Median** **(25-75 percentiles)**	47* (33-61)	52* (36-66)	70* (55-79)	66* (51-77)	63 (47-76)
**LOS, days. Median (25-75 percentiles)**	7* (5-8)	4* (3-6)	6* (4-8)	5* (3-7)	5 (4-7)
**Readmission** **(%)**	41 (4.9)	788 (98.0†)	484 (62.5)	3630 (76.3)	4943 (58.7)
**Cholecystectomy (%)**	834 (100)	804 (100)	301 (30.8)	2774 (47.7)	4713 (56.0)

Group 1- Cholecystectomy during index stay. Group 2- No cholecystectomy at index stay but within 30 days of index admission. Group 3- Sphincterotomy but not cholecystectomy within 30 days of index admission. Group 4- Neither cholecystectomy nor sphincterotomy within 30 days of index admission.

LOS = Length of hospital stay at index admission.

Readmission = Patients with at least one readmission with biliary diagnosis including acute pancreatitis within one year of index admission.

Cholecystectomy = Number of cholecystectomies within one year of index admission.

* Differences between groups, p < 0.001.

† Two percent of patients were readmitted for cholecystectomy without biliary diagnosis.

**Figure 1:**
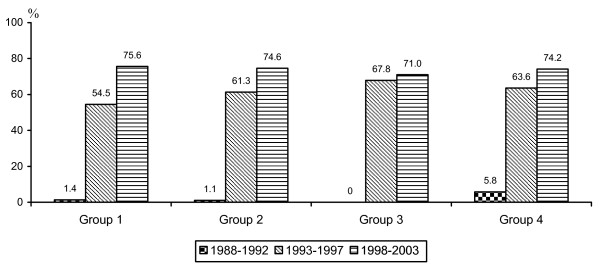
**Proportion of laparoscopic cholecystectomy in the four different MABP groups for different time periods**. Group 1 Cholecystectomy during index stay. Group 2 No cholecystectomy at index stay but within 30 days of index admission. Group 3 Sphincterotomy but not cholecystectomy within 30 days of index admission. Group 4 Neither cholecystectomy nor sphincterotomy within 30 days of index admission. Proportion in percent.

Within one year of index admission all patients in groups 1 and 2 had cholecystectomy, compared to 30.8% of patients in group 3, and 47.7% of patients in group 4. Of all patients with MABP 56.0% had undergone cholecystectomy (61% with laparoscopic technique) within one year after index admission. As demonstrated in Figure 1, the proportion of laparoscopic procedures in all groups together during the periods 1988-1992, 1993-1997, and 1998-2003 were 4.5%, 62.0% and 74.2%, respectively.

Table 2 shows that CFR within 90 days of index admission did not differ between groups 1 and 2, or between groups 3 and 4. However, CFR was significantly higher in groups 3 and 4 compared to groups 1 and 2 (p < 0.001). For all patients considered together, mortality within 90 days of index admission was significantly elevated above that of the background population, SMR 1.57 (1.42-1.75), without intergroup differences.

**Table 2:** Standardized mortality ratio (SMR) and case fatality rate (CFR, deaths/100) within 90 days of index admission in the four different MABP groups

	Group 1	Group 2	Group 3	Group 4	All groups
SMR median * (CI 95%)	1.74 (0.87-3.11)	1.38 (0.73-2.36)	1.45 (1.08-1.90)	1.60 (1.42-1.80)	1.57 (1.42-1.75)
Deaths ** (CFR)	11 (1.32)	12 (1.49)	49 (5.01)	283 (4.87)	355 (4.22)

Group 1 - Cholecystectomy during index stay. Group 2 - No cholecystectomy at index stay but within 30 days of index admission. Group 3 - Sphincterotomy but not cholecystectomy within 30 days of index admission. Group 4 - Neither cholecystectomy nor sphincterotomy within 30 days of index admission.

* SMR: No significant difference between groups.

** Deaths: Differences between group 1+2 and 3+4, p < 0.001.

Table 3 gives number (and percentages) of patients in each group versus time during the audit period. As can be seen, significantly more patients had cholecystectomy (group 1) or sphincterotomy (group 3) at index stay during the latter part of the audit. The increase was more pronounced for sphincterotomy than for cholecystectomy, and in 1998 - 2003 only 11.7% had cholecystectomy during index stay.

**Table 3:** Number of patients in the four different MABP groups in different time periods

Period	Group 1	Group 2	Group 3	Group 4	All groups
**1987-1992**	72 (6.5)	88 (8.0)	40 (3.6)	901 (81.9)	1101 (100.0)
**1993-1997**	272 (8.7)	279 (8.9)	299 (9.6)	2277 (72.8)	3173 (100.0)
**1998-2003**	490 (11.7)	459 (11.0)	638 (15.2)	2604 (61.1)	4191 (100.0)

**1987-2003**	**834 (9.9)**	**826 (9.8)**	**977 (11.6)**	**5782 (68.7)**	**8419 (100.0)**

Group 1 - Cholecystectomy during index stay. Group 2 - No cholecystectomy at index stay but within 30 days of index admission. Group 3 - Sphincterotomy but not cholecystectomy within 30 days of index admission. Group 4 - Neither cholecystectomy nor sphincterotomy within 30 days of index admission. Percent within brackets.

## Discussion

Of all patients with MABP, 9.9% had cholecystectomy at index stay and 9.8% interval cholecystectomy within 30 days of index admission. These patients were younger than patients without cholecystectomy within 30 days of index admission. Cholecystectomy at index stay slightly prolonged this stay. However, only 4.9% of these patients needed another hospital visit for a biliary diagnosis during the subsequent year as compared to 98.0% in group 2, 62.5% in group 3, and 76.3% in group 4.

We have used nation wide and validated register data based upon personal identification numbers unique for each citizen, which makes it possible to follow individuals over time and to record diagnoses and procedures during each hospital stay. As in all register studies we were provided with limited information on patients' clinical status. Aetiological classification of our patients is based upon diagnoses at index stay [3]. The Atlanta classification of MABP is not possible to use, as it is requests more specific clinical data than available in register data [11]. In accordance with previous studies [8] the definition used in our study classifies 74% of all identified patients with acute biliary pancreatitis as having mild disease.

In the audit of the present study, cholecystectomy during index stay was shown to be advantageous for the patient (one convalescence period) and for health care cost (one hospital stay). Nevertheless, only one-tenth of our patients received this alternative, and 30.8% of all patients who underwent sphincterotomy within 30 days of index admission had cholecystectomy within one year of index admission. Our finding that the majority of Swedish patients with first attack of MABP underwent gallbladder surgery after the index stay (necessitating a new admission and additional convalescence) concurs with experience from the United Kingdom [12], where delay of cholecystectomy was found to be associated with a high readmission rate [13]. According to a questionnaire study, only 58% of consultants preferred early cholecystectomy (at index stay or within 4 weeks of index admission) for MABP [14] although it is safe to proceed to cholecystectomy as soon as the serum amylase-level has started to decline [15,16]. Similar deviations from the UK guidelines [6] have been reported from Spain [17], Germany [18], Italy [19] and the US [20]. In one Swedish study [21] comprising 96 patients with gallstone pancreatitis, high age (median 74 years) and long observation time (median 84 months), 64 patients underwent sphincterotomy. However, 51 patients ultimately had cholecystectomy and out of 25 patients alive at the end of the audit period, 9 had biliary symptoms. The authors concluded that routine cholecystectomy should be considered in fit patients following acute gallstone pancreatitis. According to a recent Cochrane review, prophylactic cholecystectomy should be offered to patients whose gallbladder remains in-situ after endoscopic bile duct clearance [22].

In the present study, patients in groups 3 and 4 (patients who had endoscopic sphincterotomy but no cholecystectomy and patients without both sphincterotomy and cholecystectomy within one month of index admission) were older than group 1 patients who had cholecystectomy within one month of index admission. The higher case fatality rate in groups 3 and 4 compared to groups 1 and 2 should be considered a consequence of this age difference as SMR did not differ significantly between the four groups [3]. Thus, increasing age raised the threshold for cholecystectomy in patients with MABP in Sweden. However, even in old and high risk patients, open cholecystectomy with intra-operative cholangiography and bile duct exploration if necessary, was found preferable to endoscopic sphincterotomy with the gallbladder left in-situ as a definitive treatment for common bile duct stones or non-severe biliary pancreatitis [23].

The reasons for the low cholecystectomy rate during and shortly after index stay for patients with MABP observed in our study are not obvious. A minority of patients might have been too frail and unfit for surgery. The lack of resources or flexibility, manifesting as difficulties in finding time in operating theatres between elective procedures or more urgent operations, might have influenced the decision to delay cholecystectomy for patients with MABP. The surgeon's expectation of a difficult cholecystectomy, including treatment of common bile duct stones, might also affect the timing of surgery. It is, however, of interest that the rate of common bile duct stones in MABP and in symptomatic cholelithiasis was found not to differ significantly [24]. Mofidi et al [25] also demonstrated the possibility to follow the UK guidelines [6] for acute pancreatitis by undertaking definitive management within two weeks of index admission in 89.7% of patients with acute gallstone pancreatitis. In that study [25] 37 out of 322 patients had ERCP and sphincterotomy, and 285 patients cholecystectomy as definitive treatment for acute gallstone pancreatitis.

## Conclusion

After an attack of mild acute biliary pancreatitis, 55% of patients in our audit had cholecystectomy within one year of index admission but only 20% within one month. Cholecystectomy during index stay slightly prolonged this stay, but drastically reduced readmissions for biliary indications.

## Competing interests

The authors declare that they have no competing interests.

## Authors' contributions

The study was planned by BS, MMH, EN and MÖ. HS participated in the design of the study and performed the statistical analyses. BS, EN and MÖ drafted the manuscript. All authors have read and approved the final the manuscript.

## Pre-publication history

The pre-publication history for this paper can be accessed here:

http://www.biomedcentral.com/1471-230X/9/80/prepub
